# A case of laparoscopic high anterior resection of rectosigmoid colon cancer associated with a horseshoe kidney using preoperative 3D-CT angiography

**DOI:** 10.1186/s40792-018-0472-x

**Published:** 2018-06-27

**Authors:** Naoki Kubo, Norihiko Furusawa, Shinichiro Imai, Masaru Terada

**Affiliations:** Department of Surgery, Nagano Prefectural Shinshu Medical Center, Suzaka, 1332, Suzakashi, Nagano 382-8577 Japan

**Keywords:** Colon cancer, Horseshoe kidney, 3D-CT

## Abstract

**Background:**

Horseshoe kidney is a congenital malformation in which the bilateral kidneys are fused. It is frequently complicated by other congenital malformations and is often accompanied by anomalies of the ureteropelvic and vascular systems, which must be evaluated to avoid iatrogenic injury. We report a case of laparoscopic high anterior resection of rectosigmoid colon cancer associated with a horseshoe kidney using preoperative 3D-CT angiography.

**Case presentation:**

A 52-year-old Japanese man with lower abdominal pain underwent lower endoscopy, revealing a type 2 lesion in the rectosigmoid colon. He was diagnosed with rectosigmoid colon cancer with multiple lung metastases and a horseshoe kidney on computed tomography (CT) scan. Three-dimensional (3D)-CT angiography showed an aberrant renal artery at the isthmus from 3 cm under the inferior mesenteric artery (IMA) branch of the aorta. Laparoscopic anterior rectal resection was performed. During the operation, the inferior mesenteric artery, left ureter, left gonadal vessels, and hypogastric nerve plexus could be seen passing over the horseshoe kidney isthmus and were preserved. The left branch of aberrant renal artery that was close to IMA was also detected and preserved.

**Conclusion:**

To prevent intraoperative misidentification, 3D-CT angiography should be performed preoperatively to ascertain the precise positional relationships between the extra renal arteries and the kidney. We always must consider anomalous locations of renal vessels, ureter, gonadal vessels, and lumbar splanchnic nerve to avoid laparoscopic iatrogenic injury in patients with a horseshoe kidney.

## Background

Horseshoe kidney is a congenital malformation in which the bilateral kidneys are fused. It is frequently complicated by other congenital malformations and is often accompanied by anomalies of the ureteropelvic and vascular systems [1]. To prevent intraoperative misidentification, three-dimensional (3D)-computed tomography (CT) angiography should be performed preoperatively to identify the precise positional relationships between the aberrant renal arteries and the kidney. Anomalous locations of renal vessels, ureter, gonadal vessels, and lumbar splanchnic nerves must be evaluated to avoid laparoscopic iatrogenic injury in patients with a horseshoe kidney. We report a case of laparoscopic high anterior resection of rectosigmoid colon cancer associated with a horseshoe kidney using preoperative 3D-CT angiography.

## Case presentation

**Fig. 1:**
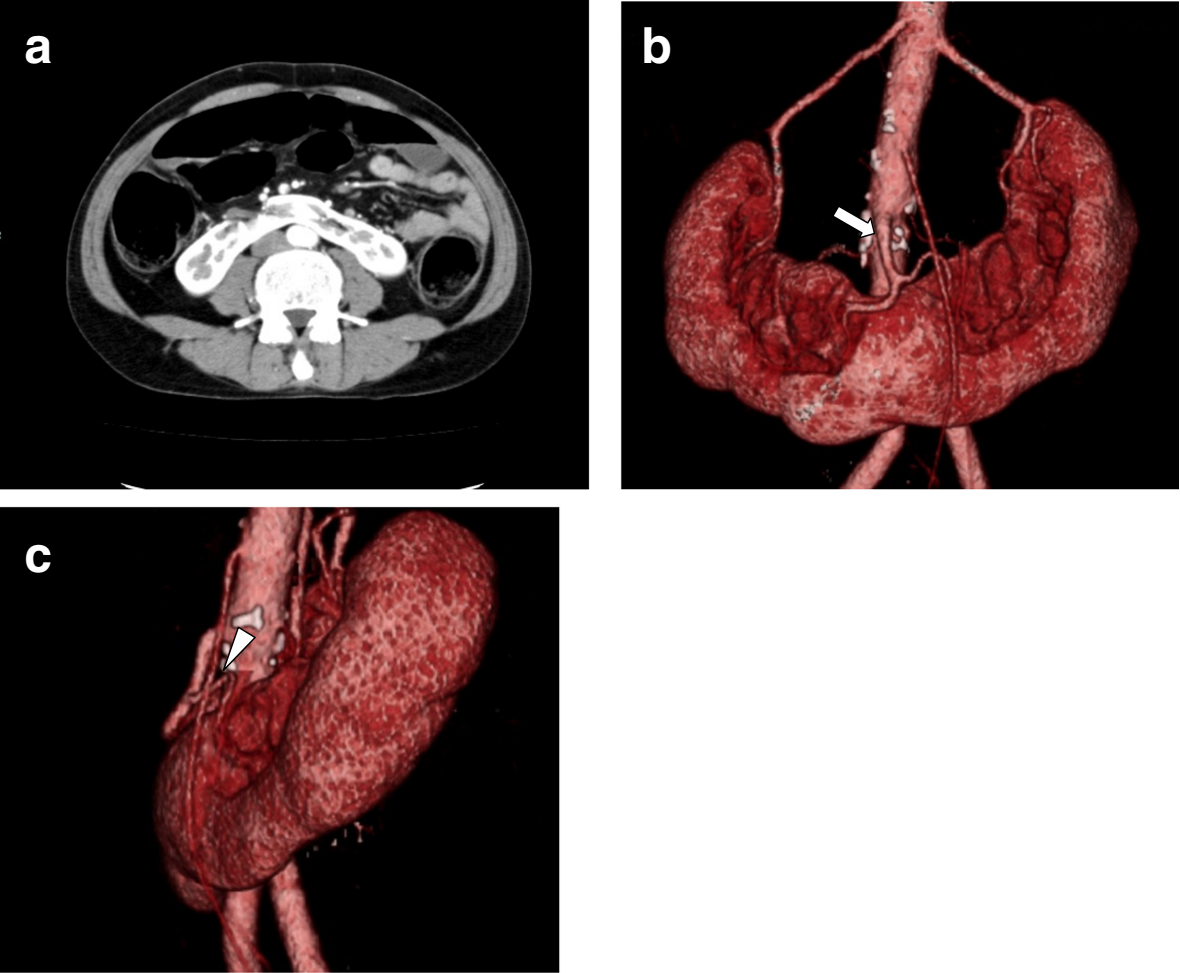
**a** Abdominal CT showing horseshoe kidney. **b**, **c** 3D-CT angiography shows the root of an aberrant renal artery (white arrow) at the isthmus from 3 cm under the IMA branch of the aorta, and the left branch of aberrant renal artery (arrowhead) was close to IMA

**Fig. 2:**
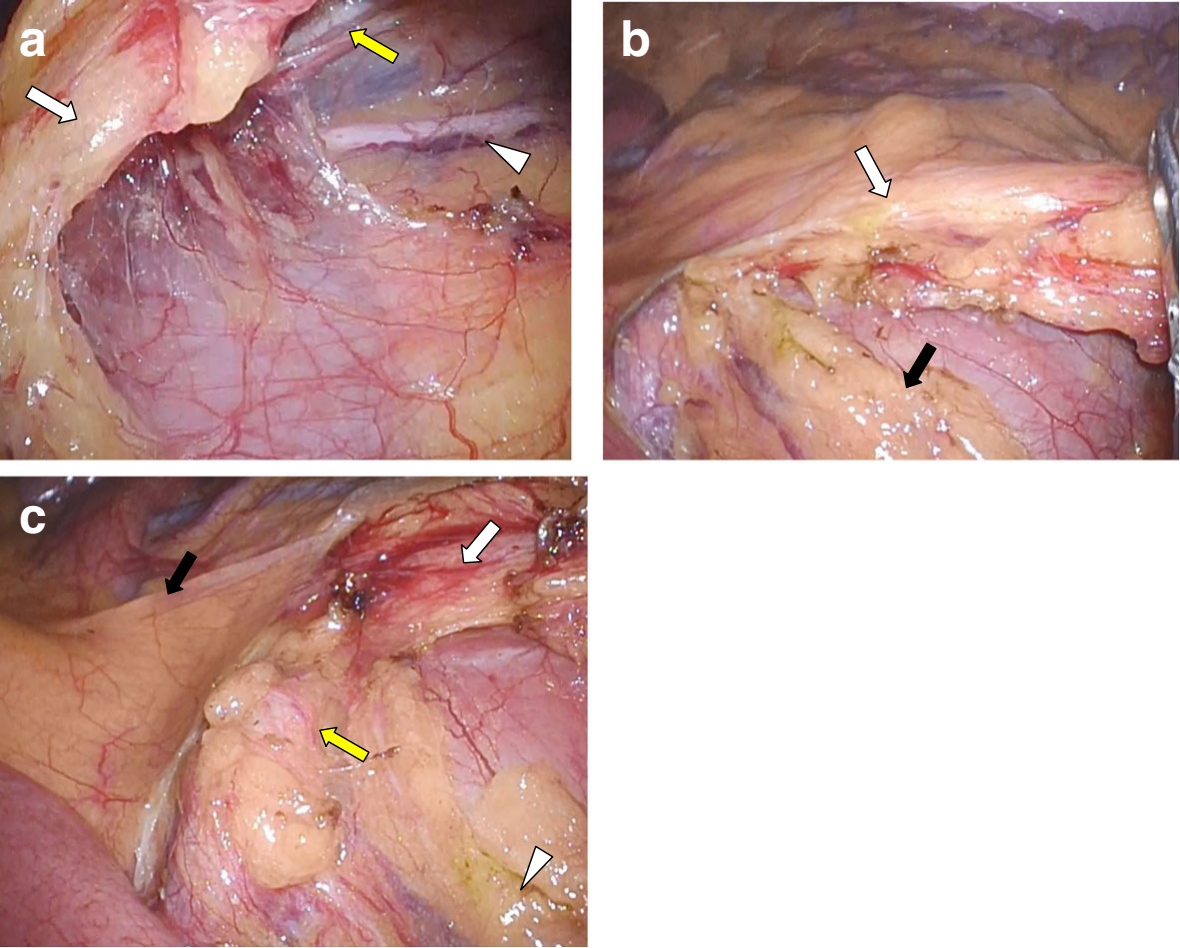
Surgical findings. **a**, **b** The IMA (white arrow), left ureter (arrowhead), left gonadal vessels (yellow arrow), and hypogastric nerve plexus (black arrow) could be seen passing over the horseshoe kidney isthmus and preserved. **c** The root of IMA (black arrow) was located considerably cephalad to the renal isthmus (arrowhead), and the left branch of aberrant renal artery (yellow arrow) was close to IMA (yellow arrow)

A 52-year-old Japanese man with lower abdominal pain underwent lower endoscopy, revealing a type 2 lesion with the entire circumference raised in the rectosigmoid colon. He was diagnosed with rectosigmoid colon cancer and underwent endoscopic stent placement as a bridge to surgery for large bowel obstruction. He was found to have multiple lung metastases and a horseshoe kidney on CT scan (Fig. 1a). 3D-CT angiography showed an aberrant renal artery at the isthmus from 3 cm under the inferior mesenteric artery (IMA) branch of the aorta (Fig. 1b). Laparoscopic anterior rectal resection was performed with a five-port conventional technique in which sigmoid colon and upper rectum were mobilized via a medial approach. During the operation, the IMA, left ureter, left gonadal vessels, and hypogastric nerve plexus were identified and preserved (Fig. 2a, b). The root of aberrant renal artery was not visualized. The root of the IMA was located considerably cephalad to the renal isthmus, and the left branch of aberrant renal artery that was close to IMA was detected and preserved (Fig. 2c). The specimen was removed through a small laparotomy wound, and intraperitoneal reconstruction was performed according to the standard double stapling technique. The patient recovered uneventfully and was discharged on postoperative day 16. Pathological examination demonstrated no metastasis of the lymph node.

### Discussion

Horseshoe kidney is rare, with an incidence of 0.25%, and the incidence is higher in males than in females at a ratio of 2:1. Horseshoe kidneys are fused at the lower pole in 95% of cases, and the isthmus is composed of fibrous tissue alone or contains parenchyma. The horseshoe kidney is located at a level lower than the normal kidneys because elevation of the kidneys interferes with the isthmus at the origin of the IMA.

A literature search revealed 23 patients who underwent surgery for colon cancer with concomitant horseshoe kidney including our patient, from 1983 to 2017 [2–6]. There were 14 men and 9 women, and the affected region was the sigmoid colon in 12, rectum in 8, descending colon in 2, and ascending colon in 1. Surgery was performed under laparoscopy in 20 patients. The positions of the anatomical structures necessary for surgery are summarized in Table 1.

**Table 1:** Summary of the positions of the anatomical structures in patients undergoing surgery for colon cancer associated with a horseshoe kidney

Ureter	Ventral aspect of horseshoe kidney: 11 cases; not available: 12 cases
Gonadal artery and vein	Ventral aspect of horseshoe kidney: 7 cases; not available: 16 cases
Lumbar splanchnic and superior hypogastric nerves	Ventral aspect of horseshoe kidney: 6 cases; dorsal aspect of horseshoe kidney: 1 case; not available: 16 cases
Extra renal artery	From the aorta: 10 cases; from the iliac artery: 7 cases; existing (details unknown): 3 cases; not existing: 3 cases; not available: 4 cases. (There is some overlap)

Regarding the ureteral distribution, it was present on the ventral side of the horseshoe kidney, i.e., almost the same as that in patients with normal kidneys, in all cases in which the distribution was described. The ureters were present on the ventral side of the horseshoe kidney in many reported cases, suggesting that it was conserved on dissection similarly in patients with normal kidneys. However, there were some cases where the ureters were present on the dorsal side of the isthmus of the horseshoe kidney, where a ureter other than the bilateral ureters independently came out from the isthmus of the horseshoe kidney, where two ureters each came out from the bilateral sides, or where the ureters ectopically opened in regions other than the urinary bladder [7], suggesting the necessity of preoperative imaging diagnosis and careful surgery.

In the cases described in the literature, the distribution of the gonadal artery and vein were usually normal. However, variations have been reported, such as the presence of two left ovarian veins and branching of the testicular artery from the renal artery and renal sinus [8], again suggesting the necessity of preoperative imaging diagnosis and careful surgery.

The bilateral lumbar splanchnic and superior hypogastric nerves were distributed on the ventral side of the horseshoe kidney in six patients reported, including in our patient, but superior hypogastric nerves distributed from the dorsal side of the horseshoe kidney into the pelvis were noted in one patient. Although there were only a few reports on the distribution of the bilateral splanchnic and superior hypogastric nerves in patients with horseshoe kidney, the second lumbar splanchnic nerves were present on the ventral side of the kidneys and the third lumbar splanchnic nerves were present on the dorsal side of the kidneys, and both nerves formed superior hypogastric plexuses in a report of an autopsied patient [6].

Arterial anomalies are reported in more than 70% of patients with horseshoe kidney, due to remain of branches from the common iliac artery, median sacral artery, and IMA. Excess renal arteries were observed in 16 of 23 patients, including patients with unclear details. Kölln classified the vascular supply in the horseshoe kidney into the following three types: (1) direct single branching from the aorta on the bilateral sides (15%), (2) several blood vessels on the bilateral sides and in the isthmus all directly branching from the aorta (65%), and (3) several blood vessels on the bilateral sides and in the isthmus that branch from common iliac artery, internal iliac artery, inferior mesenteric artery, or median sacral artery (20%) [9]. The second pattern was noted in our patient. 3D-CT angiography is useful in these cases [3, 4]. As the resolution of CT increased, the positional relationship among the IMA, kidneys, and excess renal arteries may be sufficiently identified using 3D-CT angiography.

In our patient, the IMA was easily detected during usual mobilization via median approach. We usually dissected the layers preserving the plexus, and the left branch of aberrant renal artery that was close to IMA was detected and preserved uneventfully, because the locations of the excess renal arteries and IMA were sufficiently identified using 3D-CT angiography. Injury to excess renal arteries by laparoscopic colectomy may be avoided by retaining accurate dissection layers, because they are present in the retroperitoneum. Even when an accurate dissection layer cannot be retained, laparoscopic colectomy may be safely performed by sufficiently evaluating images and confirming the anatomical structures before surgery.

## Conclusions

To prevent intraoperative misidentification, 3D-CT angiography should be performed preoperatively to ascertain the precise positional relationships between the extra renal arteries and the kidney. We always must consider anomalous locations of renal vessels, ureter, gonadal vessels and lumbar splanchnic nerve to avoid laparoscopic iatrogenic injury in patients with a horseshoe kidney.

## Abbreviations

3D: Three-dimensional
CT: Computed tomography
IMA: Inferior mesenteric artery
